# Size, Composition, and Support-Doping Effects on Oxygen Reduction Activity of Platinum-Alloy and on Non-platinum Metal-Decorated-Graphene Nanocatalysts

**DOI:** 10.3389/fchem.2019.00610

**Published:** 2019-09-19

**Authors:** Tamara Lozano, Rees B. Rankin

**Affiliations:** Department of Chemical Engineering, Villanova University, Villanova, PA, United States

**Keywords:** DFT, volcano plot, oxygen reduction (ORR), electrochemistry, metal-decorated-graphene

## Abstract

Recent investigations reported in the open literature concerning the functionalization of graphene as a support material for transition metal nanoparticle catalysts have examined isolated systems for their potential Oxygen Reduction Reaction (ORR) activity. In this work we present results which characterize the ability to use functionalized graphene (via dopants B, N) to upshift and downshift the adsorption energy of mono-atomic oxygen, O* (the ORR activity descriptor on ORR Volcano Plots), for various compositions of 4-atom, 7-atom, and 19-atom sub-nanometer binary alloy/intermetallic transition metal nanoparticle catalysts on graphene (TMNP-MDG). Our results show several important and interesting features: (1) that the combination of geometric and electronic effects makes development of simple linear mixing rules for size/composition difficult; (2) that the transition from 4- to 7- to 19-atom TMNP on MDG has pronounced effects on ORR activity for all compositions; (3) that the use of B and N as dopants to modulate the graphene-TMNP electronic structure interaction can cause shifts in the oxygen adsorption energy of 0.5 eV or more; (4) that it might be possible to make specific doped-graphene-Ni_*x*_Cu_*y*_ TMNP systems which fall close to the Volcano Peak for ORR. Our results point to systems which should be investigated experimentally and may improve the viability of future fuel cell or other ORR applications, and provide new paths for future investigations of more detail for TMNP-MDG screening.

## 1. Introduction

The use of advanced, specifically-engineered paired catalyst-support systems is likely the key to advancing the field in pursuit of several transformational technologies related to reduction of small molecules such as O_2_, CO_2_, and N_2_ (Nørskov et al., 2004; Gasteiger et al., 2005; Ferguson et al., 2012; Wang C. et al., 2012; Zhu and Dong, 2013; Staszak-Jirkovský et al., 2016; Chaves et al., 2017; Zhu et al., 2017; Halder et al., 2018; Hussein and Johnston, 2018; Lozano and Rankin, 2018; Rankin and Lozano, 2019). Such reactions are critical to meet rising global concerns in energy, the environment, and sustainability/green chemistry all driven by rising populations and associated demands for increases in the standard-of-living. In recent years graphene and graphene-metal hybrid catalyst systems have gathered growing attention in the literature (Yoo et al., 2009; Cabria et al., 2010; Compton and Nguyen, 2010; Dong et al., 2010; Bai et al., 2011; Biswas and Lee, 2011; Blonski and Hafner, 2011, 2012; Granatier et al., 2011; Lim and Wilcox, 2011; Martin et al., 2011; Ramasubramaniam et al., 2011; Sargolzaei and Gudarzi, 2011; Thapa et al., 2011; Wen et al., 2011, 2017; Yang et al., 2011; Zhao et al., 2011; Fampiou and Ramasubramaniam, 2012; Huang et al., 2012a,b; Liang et al., 2012; Liu et al., 2012; Medeiros et al., 2012; Porter and Stroud, 2012; Rudenko et al., 2012; Shigeaki et al., 2012; Siburian and Nakamura, 2012; Wang Z. et al., 2012; Wu et al., 2012; Chang et al., 2013; Chen et al., 2013, 2014; Cunci et al., 2013; Donati et al., 2013; Eelbo et al., 2013; Guo C. X. et al., 2013; Lin et al., 2013; Liu L. et al., 2013; Liu M. et al., 2013; Liu X. et al., 2013; Pumera and Wong, 2013; Su et al., 2013; Tiido et al., 2013; Xu et al., 2013; Zheng et al., 2013, 2014; Zhu and Dong, 2013; Zhu et al., 2013; Bian et al., 2014; Boukhvalov et al., 2014; Fazio et al., 2014; Fei et al., 2014; Jiao et al., 2014; Kavan et al., 2014; Kong et al., 2014; López et al., 2014; Sahoo et al., 2014; Xia et al., 2014; Xie et al., 2014; Yan et al., 2014; Jin et al., 2015; Kumar et al., 2016; Jeon et al., 2017; Klechikov et al., 2017; Rêgo et al., 2017; Tang et al., 2017). In this sense, graphene as a specific catalyst has been investigated for OER, HER, NRR, ORR activity, and more. {OER = oxygen evolution, ORR = oxygen reduction, HER = hydrogen evolution, NRR = nitrogen reduction reactions} More importantly however, graphene has been shown to be capable of further functionalization or doping for use as a support to anchor small sub-nanometer to nanometer-plus sized transition metal nanoparticle catalysts (Martin et al., 2011; Sargolzaei and Gudarzi, 2011; Huang et al., 2012b; Liang et al., 2012; Siburian and Nakamura, 2012; Chen et al., 2013, 2014; Cunci et al., 2013; Liu X. et al., 2013; Kong et al., 2014; López et al., 2014; Sahoo et al., 2014; Jin et al., 2015; Klechikov et al., 2017; Rêgo et al., 2017; Lozano and Rankin, 2018; Rankin and Lozano, 2019).

This manuscript presents striking new results that extend our initial work from the last 2 years to examine the role of sub-nanometer transition metal nanoparticles (TMNP) on graphene as metal-decorated-graphene (MDG; Lozano and Rankin, 2018; Rankin and Lozano, 2019). TMNP-MDG serves as a conceptual physical regime bridge between single-atom-catalysts (typically embedded in graphene/etc) and traditional small TMNP with large degree of faceting related to low Miller Index surfaces. Specifically, in this new work presented in this manuscript, we show the ability of dopants in the graphene support to significantly modulate (upshift or downshift) the adsorption energy of mono-atomic oxygen on 4-atom, 7-atom, and 19-atom TMNP of various binary alloy/intermetallic compositions. Oxygen (and hence, Hydroxyl) are key intermediates in the ORR and the adsorption of mono-atomic oxygen, O*, on the catalyst surface is typically given as the descriptor necessary to predict the performance of a TMNP for the ORR (Nørskov et al., 2004; Gasteiger et al., 2005; Wang C. et al., 2012; Lozano and Rankin, 2018; Rankin and Lozano, 2019).

Our prior work identified a Volcano Plot for the ORR on undoped TMNP-MDG where none of the 4-atom(A_3_B_1_) binary intermetallic systems fell on the Volcano maximum, and no systems close to the maximum used constituent elements that are significantly more abundant/affordable/sustainable than platinum or the other platinum-group-metals (PGM). Our prior work showed that there is an unanticipated inversion in the thermodynamics of OH* formation at the Volcano crossing from the “base metals” to the “precious metals.” This qualitative inversion of the anticipated ORR activity for 4-atom TMNP-MDG catalysts prompted the work in this manuscript to investigate both if this was a “quantum” size effect specific to 4-atom TMNP-MDG, and whether this effect could be further enhanced or exploited by changing the bandstructure of the graphene support through doping. The hypothesis being that a change in the graphene bandstructure supporting the TMNP catalyst would become coupled to the d-band of the TMNP and hence change its d-band center. This is in the spirit of early ORR work where alloying of “dopant” metal atoms into Pt is used to shift its *d*-band center and make it a more viable ORR catalyst, closer to the predicted maximum on the single-crystal ORR Volcano Plot (Nørskov et al., 2002, 2004; Chorkendorff and Niemantsverdriet, 2003; Bligaard et al., 2004; Gasteiger et al., 2005; Jacob and Goddard, 2006; BERNA et al., 2007; Lima et al., 2007; Greeley et al., 2009; Keith et al., 2010; Debe, 2012; Wang C. et al., 2012; Guo S. et al., 2013; Wu and Yang, 2013; Hernandez-Fernandez et al., 2014; Jackson et al., 2014; Zhang et al., 2015; Staszak-Jirkovský et al., 2016; Jinnouchi et al., 2017; Negro et al., 2017; Li et al., 2018; Wang et al., 2018).

Our new results in this manuscript show that indeed it is possible to use doped-graphene as a support which can up- or down-shift the related adsorption energy of mono-atomic oxygen of 4-, 7-, and 19-atom binary/intermetallic TMNP-MDG systems for the ORR. This upshift/downshift is reflected by a lateral change in the position of the given TMNP-MDG system on the Volcano Plot. Results from the work described in this manuscript show that the effect is highly non-linear, at least for the systems studied to date. However, because of this non-linearity, we have identified candidates for TMNP-MDG including 19-atom Au-Pd (Au-Pd is thought to be a good selective peroxide catalyst at larger catalyst particle sizes), and a new 7-atom **Ni**_*x*_**Cu**_*y*_ catalyst system which are both predicted to perform very close to the Volcano Peak if they can be synthesized (Rankin and Greeley, 2012; Staszak-Jirkovský et al., 2016). Interestingly both fall on the “opposite” side of the Volcano Peak than one might anticipate from single-crystal catalyst studies (too oxophilic or too oxophobic, respectively). The results in this work point to future directions of study including variation of the Cu-Ni system to include other compositions (*ternary metal*?), particle size, additional dopants in the graphene and more. We hope the results presented in this work will ultimately assist in the design of non-PGM (PGM = Platinum Group Metal) based, cycle-stable, ORR catalysts which can be synthesized more easily than their sub-nanometer counterparts reported in this work. Because some effects observed in this work may be due to purely quantum-size effects of the sub-nanometer catalyst(s) studied, and some of the systems discussed in this manuscript may prove challenging to synthesize, our results point to the difficult yet necessary ultimate task of extending this work far enough to identify predictive non-linear mixing rules that combine the geometric and electronic effects of the compositions and sub-nanometer particle sizes studied to guide further catalyst screening outside of the Cu-Ni system. Although such challenges lie ahead as extension of this work, this work helps pave new paths to hopefully discover a future where ORR catalysts can contribute to the fuels/energy economy without the need for as many, if any, platinum/PGM constituents.

## 2. Methods

### 2.1. Procedure and System Model

Previous work focused on identifying the thermodynamically stable 4-atom graphene-supported nanoparticle structures and calculating the adsorption energies of the intermediate species for the ORR (O*, OH*, OOH*, and O_2_*) (Lozano and Rankin, 2018; Rankin and Lozano, 2019). Following the approach from, these adsorption energies were transformed into catalytic activities and an electrochemical volcano plot was created, allowing to identify the best graphene-supported 4-atom intermetallic nanoparticles (Nørskov et al., 2004; Gasteiger et al., 2005; Gasteiger and Marković, 2009; Rankin and Greeley, 2012; Wang C. et al., 2012; Antolini, 2018; Lozano and Rankin, 2018; Rankin and Lozano, 2019). In this work, those best candidates were further studied for size and composition dependence, by increasing the number of atoms to 7 and 19 (considered to have higher stability than other particle sizes; Sakurai et al., 1999; Chaves et al., 2017; Jena and Sun, 2018; Lozano and Rankin, 2018; Gilmour and Gaston, 2019; Rankin and Lozano, 2019). The composition of these particles was kept close to the original 4-atom ones, by using a ratio of 5:2 in the 7 atom particles, and 14:5 in the 19 atom nanoparticles. Previously, bulk *AB* type alloys for each composition were created with periodic crystals and “guest” atoms spaces to maximize their distance from each other (Lozano and Rankin, 2018; Rankin and Lozano, 2019). Four, Seven, and Nineteen atom clusters were extracted from the crystalline bulk; those clusters were adsorbed onto graphene (and doped graphene) with their most electronegative -atom -rich “plane” closer to graphene.

**Figure 1:**
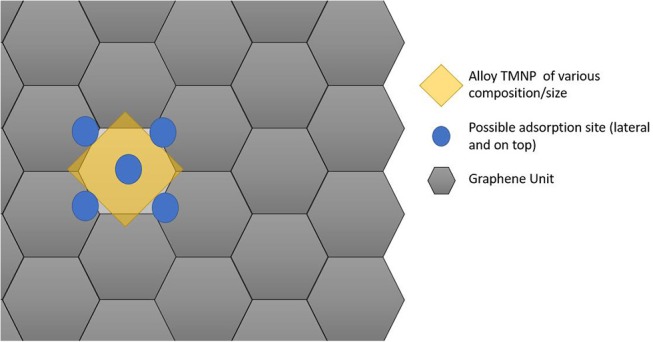
Schematic cartoon of procedure for studying adsorption of mono-atomic oxygen on TMNP-MDG for various alloy/intermetallic compositions and particle sizes. For a given TMNP composition/size, the 5 distinct lateral/atop site M3 faces above the graphene plane were studied. Results are tabulated and reported in full in the Supporting Information. Key selected results are presented and discussed in the remainder of the manuscript.

**Figure 2:**
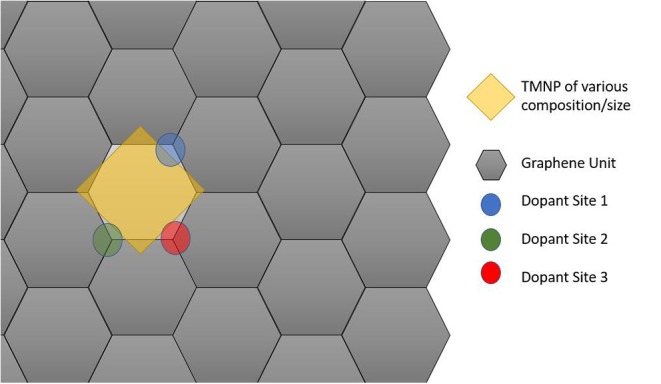
Schematic cartoon of procedure for studying adsorption of mono-atomic oxygen on TMNP-doped-MDG. Sites of the dopants studied are indicated in Blue, Green, and Red. If the TMNP spanned 2 Graphene units the vertex closest to the outside of the perimeter of the TMNP was used as the position of the doping element(s).

Since the particles are increasing in size, the size of the graphene unit cell also needed to be increased to ensure no interactions between adjacent active sites in the lateral direction of the graphene plane. For these set of calculations, a sheet of graphene containing 128 carbon atoms was created with dimensions 17.126 Å by 19.776 Å. The newly created nanoparticles were placed ~2.5 Å over the new graphene sheet, centered over hexagonal rings creating the new catalytic structures. Structure optimizations were run in MedeA-VASP allowing all atom geometries to relax until finding the local structure with minimum energy. Schematic diagram cartoons for the representative structure/procedures for studying the mono-atomic O* adsorption on undoped (Figure 1) and doped (Figure 2) graphene are given as Figures 1, 2, respectively. Structure graphics are provided in the Supporting Information for the best candidate materials identified in this work.

A similar approach to that described in our previous work was used for the size and dopants studies on transition metal nanoparticle (TMNP) decorated graphene (MDG; Lozano and Rankin, 2018; Rankin and Lozano, 2019). Plane-wave density functional theory (DFT) calculations are carried out using the Vienna *Ab Initio* Simulation Package (VASP) within the MEDEA Simulation environment, since they are well-suited to modeling periodic highly-symmetric structures (Kresse and Hafner, 1993a,b, 1994; Kresse and Furthmüller, 1996a,b; Materials Design, Inc., 2016; Lozano and Rankin, 2018) The specific procedural sequence of calculations and analysis was as follows:

1. Study of the adsorption of mono-atomic oxygen (O*) on the 7 and 19 atom nanoparticles, considering up to 5 different high symmetry lateral/top sites, to study how the different intermetallic faces would interact with oxygen and study the effect of geometrical and electronic effects. The initial distance between the alloy nanoparticle and the oxygen atom was initiated at 2.5 Å.
2. Adsorption energies of oxygen between the different sized particles were compared, and the “size-effect” on the oxygen adsorption energy was calculated, leading to the positions of these bigger nanoparticle systems in the volcano plot.
3. Introduction of dopants in a pure graphene structure, to study the effect on its electronic structure and the possible introduction/modulation of the graphene band gaps.
4. Study of the 4, 7, and 19 nanoparticles adsorbed on B and N doped-graphene, by substituting the carbon atoms closer to the nanoparticle by the selected dopant. For the 4-atom particles, only one dopant at a time was introduced, in 6 different positions closer to the nanoparticle to determine if the proximity to the adsorption site is a factor to be considered when using dopants in a catalyst. For the 7-atom nanoparticles, studies included the introduction of 1 and/or 2 dopants. The 2 dopants were set so that there were at least two carbon atoms in between the two doped sites. For the 19-atom particles, 1, 2, and 3 dopants were introduced, in the latter case leaving only 1 carbon atom in between doped sites.
5. Study of the adsorption of atomic oxygen (O*) on the doped systems, considering only the lowest energy high symmetry lateral site position(s).
6. Adsorption energies of oxygen between the doped and non-doped systems were compared, and the “dopant-effect” on the oxygen adsorption energy was calculated, leading to the positions of doped systems in the volcano plot.
7. The best identified size-dopant-composition NP supported on graphene was identified as having a descriptor value nearest the volcano plot maximum and recommended for experimental validation or further investigation.

For the size studies, energy minimization calculations were done to find the system's lowest energy adsorption site structure through a self-consistent iterative process. For the case of the doping studies with adsorbed graphene, structure minimizations were performed only for the 4-atom nanoparticles; while pre-optimized (no dopant) and then refined single point (with dopant) calculations provided the energetic values for the bigger-sized systems with 7 and 19 atom nanoparticles. Our initial calculations on this subset show that, for structures with a single sheet of graphene, the use of a single point calculation vs. a full structure optimization generates a relative change in the magnitude of the adsorption energy less than 0.20%. Considering our goal in this work is identification of relative energies for our descriptor, not absolute adsorption energies, this level of change in accuracy is deemed acceptable for materials screening purposes.

### 2.2. DFT Calculations

For all the calculations described in this study, the opt-PBE functional was selected, as it shows high accuracy for the calculation of adsorption energies on MDG (Andersson et al., 1996; Perdew et al., 1996; Dion et al., 2004; Hafner, 2008; Klimeš et al., 2010, 2011; Lim et al., 2011; Berland et al., 2015; Gautier et al., 2015; Lozano and Rankin, 2018; Rankin and Lozano, 2019). Because graphene-based systems often suffer from London-Dispersion stabilized attractions on non-defect sites, the functionals to be used in adsorption energy calculations on metal-decorated graphene (MDG) often include a Van der Waals correction (Samuel et al., 2012; Lazar et al., 2013; Tim et al., 2013; Zeller et al., 2014; Praveen et al., 2015; Karlický et al., 2017). Therefore, all the calculations were done with the Van der Waals corrected opt-PBE functional at the gamma-centered *K-*point, with a Methfessel-Paxton Fermi smearing of 0.2 eV, and with convergence of the electronic ground state to energies less than 10^−5^ eV/mole-unit cell (Methfessel and Paxton, 1989). The structural optimizations were performed until the total force on all atoms relaxed were below a net magnitude of 0.035 eV/Å. All the surface cells contained at least 20 Å of vacuum (corresponding to at least 83% of total vertical distance {z} of calculation cell model). Surface supercells were composed such that adjacent metal NP (of all sizes) were accordingly separated by at least 6.5 Å in the in-plane directions; these treatments are consistent with or exceed our previous efforts and reports (Lozano and Rankin, 2018; Rankin and Lozano, 2019).

The energy of gas-phase O_2_ is generally poorly reproduced by GGA-based DFT methods, so its energy was adjusted to reproduce the tabulated gas phase O_2_ dissociation energy the tabulated thermochemistry for the formation enthalpy of water (Nørskov et al., 2004; Mehmood et al., 2012; Rankin and Greeley, 2012; Wang C. et al., 2012; Kulkarni et al., 2018; Lozano and Rankin, 2018; Rankin and Lozano, 2019). Accounting for this error, the magnitude of that correction was found to yield an absolute error of 0.102 eV/mole, or 0.051 eV/mole O* when using the DFT energy for H_2_O in the calculation of the enthalpy of formation. We have further upshifted the relative energy of our reactant system accordingly. Dipole corrections were applied to all of our slab-based calculations of MDG model systems. The magnitude of this correction was consistently found to be an average of 0.073 eV/mole per supercell with calculation cells using over 20 Å of vacuum spacing for the systems studied, much less than 0.1% of the total energy of the system and insufficient to significantly bias any conclusions presented for relative energies in this manuscript.

## 3. Results and Discussion- Effects of NP Size on TMNP-MDG Catalytic Activity for ORR

### 3.1. Energetics of Bare TMNP-MDG Systems

**Figure 3:**
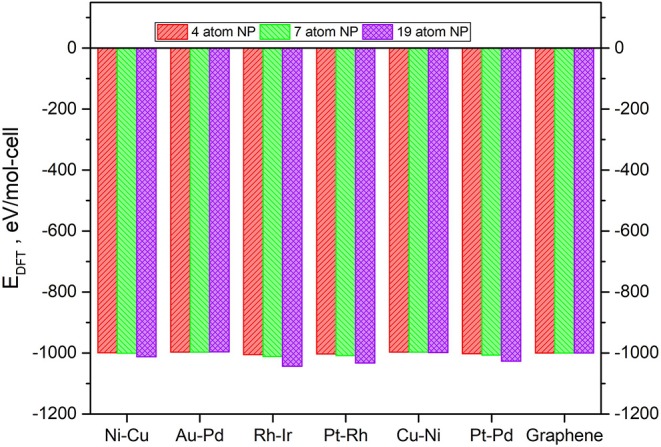
Ground-state total energy for adsorbed TMNP-MDG vs. graphene for each of the composition and size TMNP-MDG highlighted as possible ORR catalyst candidates in this manuscript.

The values of the ground state total energies for the 7 and 19-atom TMNP-MDG are reported in Figure 3, with the energies of the graphene sheet and corresponding stoichiometric amount of TMNP included as the reference state. The energy of the 4-atom particles has also been included for comparison, but it has been scaled and adjusted for the bigger graphene sheet since the prior work was performed with smaller calculation cells in the lateral (in-plane of the surface) direction(s) (Lozano and Rankin, 2018; Rankin and Lozano, 2019). All the calculations concluded with moderately to strongly negative changes to the system total energy upon adsorbing a TMNP on the graphene. This energetic sign convention is an indication that these systems could theoretically be stable if they can be synthesized. In general, the ground state energies decrease strongly non-linearly with size (# of atoms in the TMNP), indicating the structures become more energetically preferred as the number of atoms constituting them increases. We discuss this in more detail in the next paragraph.

**Figure 4:**
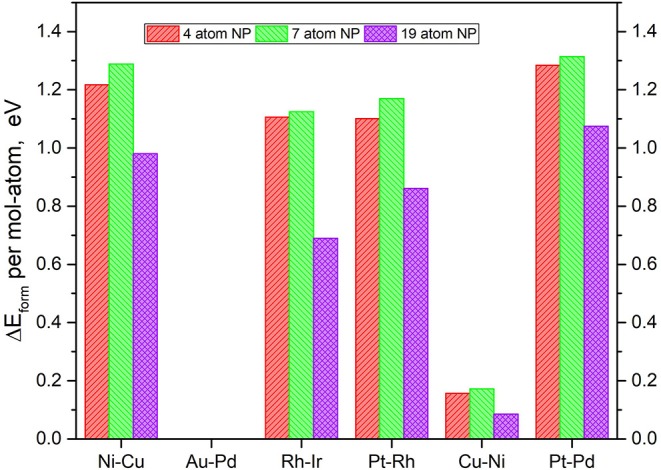
Relative DFT formation energy, in eV per atom-mol of elemental metal, for each of the composition and size TMNP-MDG highlighted as possible ORR catalyst candidates in this manuscript.

It is imperative to also consider the actual atom-by-atom thermodynamics of the TMNP-MDG system compared to the bulk elemental structure of the system's constituent elements. The calculation of the formation energies of the graphene-supported nanoparticles was calculated as the energy resulting from the adsorption of the alloys, as shown in Equations (1) and (2). It is worth noting at this point that all of the metal reference energies studied in this section come from metals calculated in the FCC crystal structure with 16 atoms per unit cell at their known lattice constant(s). Figure 4 shows the resulting formation energies normalized to a per-atom basis, to study the size effect.

For the 7 atom TMNP-MDG and the 19 atom TMNP-MDG, respectively

(1) $$\begin{array}{r} \Delta E_{form}=E_{M\;DG\;system}-\frac{7}{16}E_{bulk\;alloy}-E_{graphene} \end{array}$$

(2) $$\begin{array}{r} \Delta E_{form}=E_{M\;DG\;system}-\frac{19}{16}E_{bulk\;alloy}-E_{graphene} \end{array}$$

In general, small, positive formation energies are necessary to form these bigger TMNP-MDG systems, as shown in the literature (Ferguson et al., 2012; Mehmood et al., 2012; Tyo and Vajda, 2015; Bliem et al., 2016; Gao et al., 2016; Luo et al., 2016; Chakraborty and Pradeep, 2017; Concepción et al., 2017; He and Jagvaral, 2017; Halder et al., 2018; He et al., 2018; Jäger et al., 2018). While increasing the size from 4 to 7 atoms implies slightly higher energy; the results show the opposite for the 19-atom NP. In all the alloy NP studied, the formation energies decrease significantly when forming 19-atom particles, showing up to 0.5 eV less required to form 19-atom Rh-Ir NP for example, as compared to the corresponding smaller Rh-Ir TMNP-MDG.

### 3.2. Adsorption of Oxygen on 7-Atom and 19-Atom TMNP-MDG Systems

**Figure 5:**
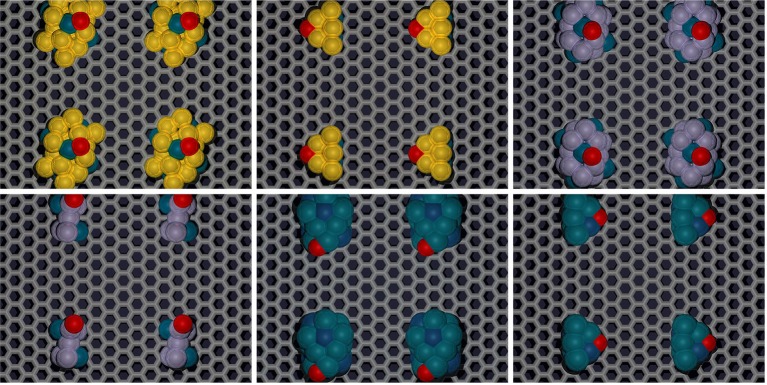
Representative 19, and 7-atom structures of select Au-Pd, Pt-Rh, and other candidate TMNP-MDG systems located “near” the Volcano Peak. Color scheme: carbon:gray, oxygen:red, TM species: yellow (Au), blue (Pd), purple (Pt), dark-blue (Rh). Results pertaining to these representative structures are explained in detail throughout the remainder of the manuscript.

**Figure 6:**
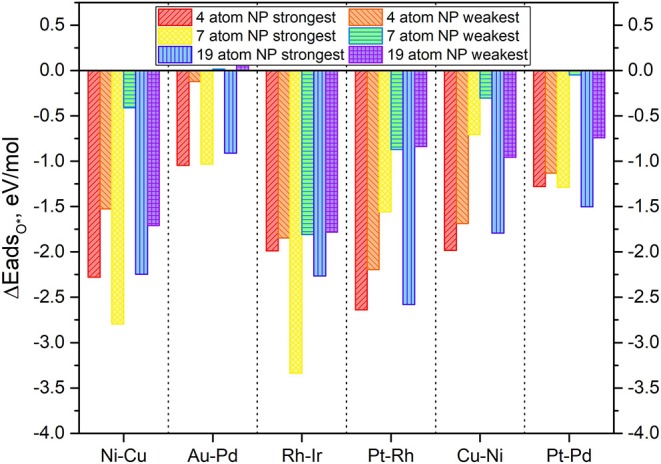
Adsorption energy of mono-atomic oxygen atoms on the 4-atom, 7-atom, and 19-atom key candidate TMNP-MDG catalysts discussed in this manuscript.

Our previous works already developed the scaling relations for small 4-atom TMNP-MDG (Lozano and Rankin, 2018; Rankin and Lozano, 2019); therefore the values for the energies of the adsorption of O* should be sufficient as a common descriptor to find the adsorption energies of other intermediate species for the oxygen reduction reaction. Structure optimizations of the larger nanoparticles were carried out with and without an oxygen atom to study how the change in size affects the adsorption energy of oxygen. Up to five different high symmetry lateral positions were studied, to study how the differences in the lateral surface sites may affect the affinity of the oxygen for these sites due either geometrical and electronic effects. The initial distance between the TMNP and the oxygen atom was set at 2.5 Å before structure-optimization. Typical structural relaxations for the work in this manuscript involved optimization of the TMNP-O bond distance to 1.9 to 2.1 Å. A representation of some of these structures/sites can be found in Figure 5 below. The detailed list of the studied structures, positions, and their resulting energies can be found in the *SI*. The adsorption energies of mono-atomic oxygen were calculated by considering the adsorption reaction of O_2_ gas on to the TMNP-MDG surface according to Equation (3). The results of the DFT based adsorption energies can be found in Figure 6, where the results obtained for the 4-atom particles have also been included for comparison.

(3) $$\begin{array}{r} \frac{1}{2}O_{2}+*↔O*\\ \Delta E_{adsO^{*}}=E_{O^{*}}-\frac{1}{2}E_{O_{2}}-E_{TMNP-MDG} \end{array}$$

In general, it is expected that as particle size increases the adsorption energies should decrease in magnitude, as the particles become less reactive. Additionally, it should be expected that increasing the amounts of an oxophobic metal, would result in a decrease in the adsorption energies of O* while increasing the amounts of oxophilic metals would result in the opposite (Nørskov et al., 2004; Gasteiger and Marković, 2009; Wang C. et al., 2012; Antolini, 2018; Lozano and Rankin, 2018; Rankin and Lozano, 2019). The obtained results in Figure 6 agree with these observations. For example, the particles with Au-Pd, see their O* adsorption energy weakened as a result of the size increase and the increase on the amount of Au (an oxophobic metal) in their composition, becoming even positive for the weakest adsorption high symmetry site. Similar results are observed with Pt-Pd TMNP-MDGs, that see the energy of the weakest adsorption site heavily modified due to the size increase, while the strongest adsorption site remains with approximately the same values of O* adsorption energies. The presentation of range of adsorption energies per catalyst composition/size is to illustrate the possible shift in descriptor values that might occur as the coverage of adsorbates increases; systems with the smallest changes from strongest to weakest adsorption sites will lie closest to their ideal Volcano position regardless of surface coverage.

Catalyst nanoparticles with Ni in their structure should see stronger adsorptions of O* due to the higher amounts of this oxophilic metal while reducing the adsorption values due to the larger sizes. When looking at the results for Ni-Cu particles, the adsorption energies have weakened (become less negative), which suggests that the geometric effect created by the change in size causes larger implications in the O* adsorption energies than the electronic effect created by the change in composition.

In general, all the TMNP-MDGs experience a similar mix of both geometric and electronic effects. The electronic effects due to the change in a composition seem to be more prominent in the 7-atom TMNP-MDGs while the geometric effects due to size take over in the 19-atm TMNP-MDGs. As a result, most of the 7-atom TMNP-MDGs become more oxophilic while the 19-atom TMNP-MDGs are generally more oxophobic. This general trend of weakened O adsorption energy with particle size should fit with established ideas of particle stability increasing as the fraction of atoms that are extremely under-coordinated relative to their bulk crystal structure decreases (which occurs as # of atoms increases in particle).

Based on these first results, there does not immediately seem that there are completely unifying “mixing” rules that allow prediction of how a TMNP-MDG will behave based on composition, which motivates our need to carry out further studies by varying the size or composition of a TMNP-MDG until the feasibility of such predictive “mixing” rules can be established more clearly.

### 3.3. Position(s) of 7-Atom and 19-Atom TMNP-MDG on the ORR Volcano Plot

The effect of the composition change can be calculated by simple numerical difference, by calculating the effect on the energy of the adsorbed TMNP-MDG without O* (Equation 4) and with O* adsorbed (Equation 5). Thus, the change in the position of the TMNP-MDG on the Volcano Plot can be calculated as the difference between these effects, following (Equation 6). Negative changes indicate a shift toward the left of the predicted maximum, while positive changes show a shift toward the right.

(4) $$\begin{array}{r} Effect_{NP}=E_{NP,new}-E_{NP} \end{array}$$

(5) $$\begin{array}{r} Effect_{O^{*}}=E_{O^{*},new}-E_{O^{*},old} \end{array}$$

(6) $$\begin{array}{r} Change_{O^{*}}=Effect_{O^{*}}-Effect_{NP} \end{array}$$

**Figure 7:**
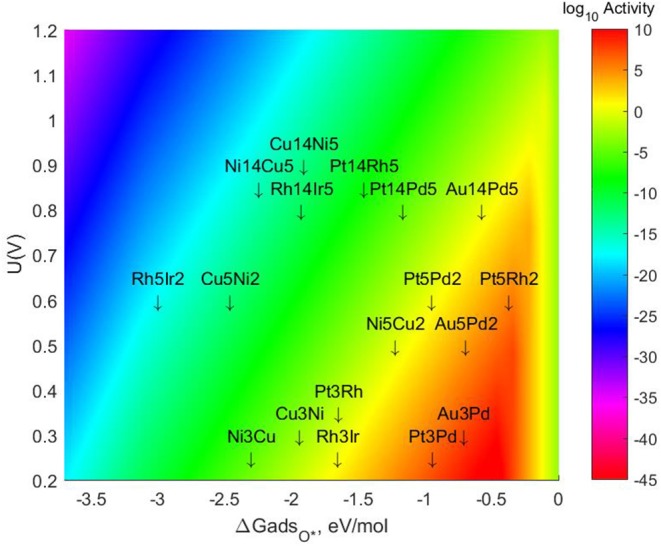
Volcano plot for ORR activity on 4-atom, 7-atom, and 19-atom TMNP-MDG reported in this manuscript.

The specific energetic results and changes in the values of mono-atomic O* adsorption energies can be found in the tables provided in the *SI*. The resulting Volcano Plot including the most stable lateral positions for these bigger TMNP-MDGs can be found in Figure 7. The final location of each of the TMNP-MDGs on the Volcano Plot results from a mixture of the geometric and electronic effects associated with the changes in size and composition.

In general terms, the 7-atom TMNP-MDG are slightly more susceptible to electronic effects (possibly due to quantum size effects), so the addition of Pt and Au shifts the alloys toward the right, while the addition of Rh or Cu shifts them toward the left. For the 19-atom NP, the geometric effect becomes more important (likely as quantum size effects decrease), and most of the particles shift toward the right due to the size increase inherently stabilizing the NP. However, there are some exceptions to these general rules, which reiterates the challenge of developing comprehensive predictive simple linear mixing-rules for these types of systems. The closest system to the predicted volcano maximum becomes Pt_5_Rh_2_, which is located 0.094 eV on the right side of the maximum. The closest system on the left side of the predicted maximum is Au_14_Pd_5_, missing the target by only 0.112 eV, followed by Au_5_Pd_2_ which is 0.233 eV on the left of the maximum. These ranges have been previously studied as the realistic target window for designing ternary intermetallic catalysts with extended single crystal surface structure (Nørskov et al., 2004; Gasteiger and Marković, 2009; Rankin and Greeley, 2012; Wang C. et al., 2012; Lozano and Rankin, 2018; Rankin and Lozano, 2019). It could be expected then that creating a ternary alloy by adding some Pt or Rh to this Au_5_Pd_2_ system would shift its location toward the right, possibly reaching the volcano maximum.

Some studies have been done with much larger particles and alloys with a similar composition of Au-Pd have been shown to near the volcano maximum for the selective synthesis of H_2_O_2_ from the left (Rankin and Greeley, 2012). Thus, it is clear that particle size can be an important factor not only in the catalytic activity but also for selectivity purposes.

### 3.4. Bandshifts in Doped Graphene Sheets

Most of the applications for graphene require the significant challenge of opening a sizable and well-tuned bandgap in it, in order to modify its electron mobility (Liu et al., 2011; Wang H. et al., 2012; Putri et al., 2015; Cheng et al., 2018; Lee et al., 2018; Kim et al., 2019; Rosli et al., 2019; Rouhani, 2019). Substitutional doping methods can be used to open the bandgap and tune the Fermi level of graphene. Boron and Nitrogen atoms are the natural candidates for doping in graphene, because of their similar atomic size as that of carbon, and their hole acceptor (boron) and electron donor (nitrogen) characters (Riedl et al., 2010; Telychko et al., 2015). In this work, substitutional doping is taken one step further, studying the substitution of carbon by seven elements: the commonly studied nitrogen and boron, but also aluminum, sulfur, silica, oxygen, and phosphorus. DFT structure optimizations were performed in MedeA-VASP for each of the systems, using the same OPT-PBE functional with the Van der Waals correction. The dopants were introduced at approximately 1 single dopant per 50–100Å^2^ of graphene to be consistent with bridging our older and current work (Gong et al., 2009; Wu et al., 2012; Lu et al., 2013; Fazio et al., 2014; Fei et al., 2014; Kong et al., 2014; Yan et al., 2014; Lozano and Rankin, 2018; Rankin and Lozano, 2019).

**Figure 8:**
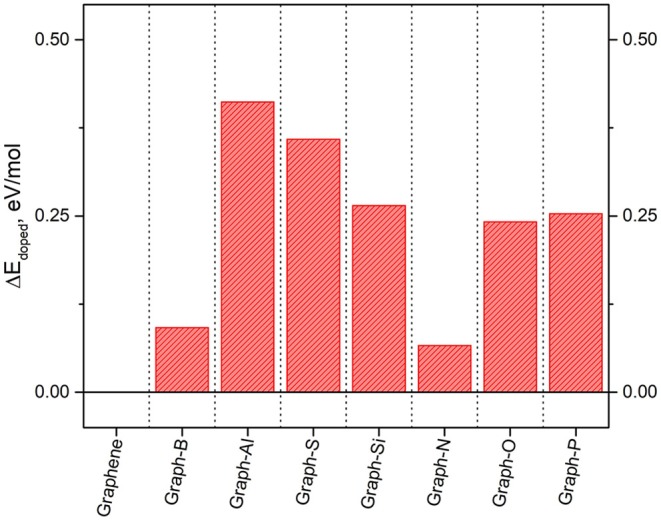
Energetic penalty, per mole of dopant atom, to insert 3% dopant loading into graphene.

The resulting ground state energies are shown in Figure 8, where the ground state energy of graphene is also included for comparison purposes. It is shown that doping decreases the ground state energy of the systems by up to ~13 eV with respects to pure graphene. The systems with the lowest values of ground state energies (the most stable ones) are those including elements in the first row of the periodic table (boron, nitrogen, and oxygen) as they are the closest in size to the original carbon atom. On the other hand, elements with a considerable difference in size with respect to carbon, result in a larger change in the ground state energy with respect to graphene. The biggest differences are observed when doping with aluminum, and they are most likely due to the metallic character of the dopant.

Results for the density of states (DOS) for some of the systems were also obtained from these calculations and some of the results have been included in Table 1. The different results on each of the dopants are directly related to the electron donor/acceptor character with respect to carbon. Electron acceptors attract the electrons, creating “holes” in the charge density of the system. Elements with more electrons than carbon become electron donors, while elements with fewer electrons become acceptors. In this case, nitrogen is an electron donor while boron is an electron acceptor.

**Table 1:** Bandgaps in V for the doped-graphene reported in this work.

**Dopant element**	**B**	**Al**	**S**	**Si**	**N**	**O**	**P**
Bandgap (eV)	0.85	1.15	0.80	0.10	0.70	0.65	1.05

As a consequence, the band structure and DOS of the system get modified, which affects the location of the Fermi level and alters the band gap. In general terms, it is expected that electron donors will maintain a small gap, resulting in slight changes in the conductivity, while electron acceptors result in larger gaps, as they withdraw significant electron density. Similar results can be observed in Table 1 for the dopants included in this study. The Fermi level and the location of the HOMO and LUMO orbitals get lowered down when nitrogen is introduced in the system and raised up when the dopant is boron. The observed shift reaches up to ~2 eV in some cases. Moreover, the presence of dopants introduces a band gap in the graphene's electronic structure. The specific values of the bandgap obtained for each of the studied dopants can be found in Table 1. The largest gap observed was 1.15 eV obtained for the case where Al is used as a dopant, but it is worth noting that is an indirect gap. In a direct band-gap, the edges are aligned, and the electrons can transfer into the conduction band without a change of momentum. An indirect bandgap does not have the edges aligned, and the electrons are only transferred into the conduction band through a shift in electron momentum (Putri et al., 2015; Rosli et al., 2019). The electron transfers included in this study are electron-bond recombination, not really energy level promotions, so the band gaps are indirect. Thus, the main conclusions from this study are the tunable electronic structure of graphene that allows to introduce an indirect band gap of ~1 eV. The dopants leading to the most stable structures are boron and nitrogen, and given their contrasting donor/acceptor character, are two elements are the first selected in our work to modify the properties of the TMNP-MDG that have been the subject of the previous studies.

### 3.5. Oxygen Adsorption on 4, 7, and 19 Atom TMNP on Doped-Graphene

The most stable configurations for O* adsorbed on the different size TMNP-MDGs were selected for the dopant studies. For the 4-atom TMNP-MDGs, only one dopant atom was introduced, by substituting for each of the 6 graphene carbon atoms closest to the TMNP-MDG alloy the “perimeter” edge(s) {(direct proximity under TMNP perimeter atoms)}. By studying different dopant positions it is possible to determine if the proximity to the adsorption site is a factor to be considered when using dopants in a catalyst. For the 7-atom TMNP-MDGs, studies included the introduction of 1 and 2 dopants at a time. The 2 dopants were set so that there were at least two carbon atoms in between the two doped sites, to reduce the distortion of the original graphene structure that would be caused by the assumed Coulombic repulsion(s). For the 19-atom particles, 1, 2, and 3 dopants were introduced, in the latter case leaving only 1 carbon atom in between doped sites.

Since the sheer volume of the results from this work are intractable for discussion individually in a single manuscript, only the most promising candidate TMNP-MDG catalyst and dopant pairings are discussed in this section. The complete set of values for the different dopant sites can be found in the corresponding tables in the *SI*. The results for the systems with the strongest adsorption energies of oxygen were assumed to be the most likely configurations and were analyzed further to determine their location on the Volcano Plot. Due to the large amounts of data in this manuscript, the results have been separated for the different alloy/intermetallic compositions of TMNP-MDGs studied. As a reminder, the energy of adsorption of mono-oxygen on the TMNP-MDG is calculated as the energy of the reaction of adsorption of O_2_ gas on to the TMNP-MDG surface, according to Equation (3) previously given. Additionally, the effect of the addition of dopants can be calculated by difference, similarly to the effect of composition change described previously. Negative changes in the position of the particles indicate a shift toward the left of the predicted maximum, while positive changes show a shift toward the right. This convention is similar to that described in the preceding subsection in Equations (4–6).

(7) $$\begin{array}{r} Effect_{doped-NP}=E_{NP,doped}-E_{NP,undoped} \end{array}$$

(8) $$\begin{array}{r} Effect_{doped-O^{*}}=E_{O^{*},doped}-E_{O^{*},undoped} \end{array}$$

(9) $$\begin{array}{r} Change_{O^{*},doped}=Effect_{doped-O^{*}}-Effect_{doped-NP} \end{array}$$

#### 3.5.1. Au-Pd TMNP on Doped Graphene

The results for the adsorption energies are included in Figure 15. In general terms, the addition of dopants in the graphene structure increases the adsorption energy of oxygen, making the O-NP bond weaker. It is significant also to consider the position of the dopants that makes the adsorption of oxygen stronger. In the systems where B is used as a dopant, the strongest adsorptions are found when the B is placed as far from the oxygen atom as possible. Since B is an electron acceptor, the TMNP-MDG experiences a deficit of electron-density that reinforces the O-NP bond. The opposite occurs when N is used as a dopant, since the strongest adsorption energies are found when the N is placed as close to the O as possible. The 4-atom TMNP-MDG experiences an adsorption of O*~0.3 eV higher when B is present than when adsorbed on pristine graphene. For bigger TMNP-MDGs, nitrogen as the dopant causes the largest effect, increasing the adsorption of O* by ~0.15 eV. In general terms, the addition of a second or third dopant atom only changes the adsorption energies by ~0.01–0.02 eV, so its effects are barely perceptible.

**Figure 9:**
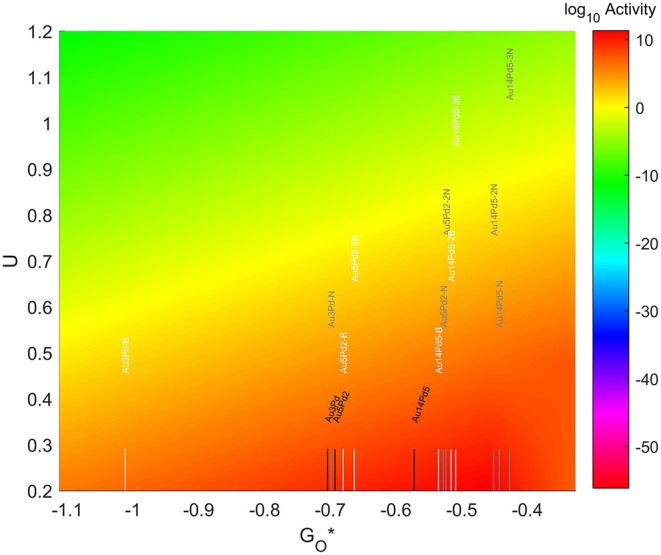
Volcano plot for ORR activity of Au-Pd 4-atom, 7-atom, and 19-atom TMNP-doped-MDG. 4/7/19 atom systems are Au_3_Pd_1_, Au_5_Pd_2_, and Au_14_Pd_5_, respectively.

The resulting Volcano Plot is shown in Figure 9, zoomed in for visual clarity. The results of the particles supported on pristine graphene have also been included for comparison. It can be observed how the addition of dopants shifts the location of the alloys on the volcano, making some of the systems become oxophobic and appearing on the right side of the volcano maximum. The best system, in this case, is Au_14_Pd_5_ with only 1 atom of N in its graphene structure, which exceeds the position of the predicted maximum by 0.0386 eV.

#### 3.5.2. Pt-Pd TMNP on Doped Graphene

The results for the adsorption energies are included in Figure 15. Again, for the 4-atom and 7-atom TMNP-MDGs, the addition of dopants in the graphene structure increases the adsorption of oxygen, making the O-NP bond weaker. However, for the 19-atom particles, the inclusion of boron in the graphene structure decreases slightly the energy of adsorption of O* concerning system with pristine graphene. Considering the position of the dopants results significantly once again, since the systems with B as a dopant produce its largest adsorptions when the B is located far away from the O, while for the N-doped systems the N-O distance is kept as small as possible.

The 4-atom TMNP-MDG experiences an adsorption of O*~0.1 eV stronger when B is present than when adsorbed on pristine graphene. For the 7-atom TMNP-MDG it's again B the dopant causing the biggest effect, with a change of almost 0.15 eV. However, when adding a second dopant into the system, the addition of boron barely modifies the structure, while the addition of a second nitrogen increases the adsorption of O* by ~0.1 eV. For the 19-atom particles, the presence of B decreases the adsorption of O* by 0.05 eV while N increases the O-NP bond strength by approximately the same amount. In this case, the addition of a second or a third dopant doesn't generate significant changes in the O* adsorption energies.

**Figure 10:**
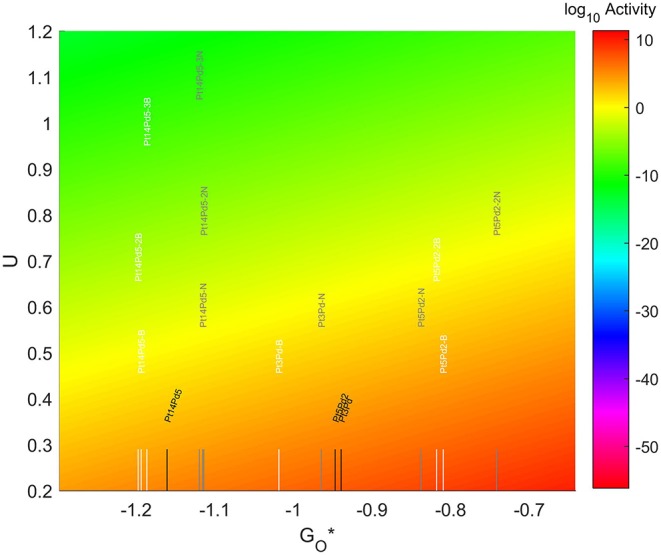
Volcano plot for ORR activity of Pt-Pd 4-atom, 7-atom, and 19-atom TMNP-doped-MDG. 4/7/19 atom systems are Pt_3_Pd_1_, Pt_5_Pd_2_, and Pt_14_Pd_5_, respectively.

The location of these TMNP-MDGs in the volcano plot is shown in Figure 10, zoomed in for visual clarity. The results of the particles supported on pristine graphene have also been included for comparison. In general terms, the addition of N dopant makes the systems more oxophobic, so they are shifted toward the right, while the addition of B tends to shift them toward the left. In this case, none of the systems becomes oxophobic enough to overcome the volcano maximum. The best system, in this case, is Pt_5_Pd_2_ with 2 atoms of N in its graphene structure, which is 0.282 eV on the left of the predicted volcano maximum.

#### 3.5.3. Pt-Rt TMNP on Doped Graphene

The results for the adsorption energies are included in Figure 15. The adsorption energies in this case have decreased as the size of the TMNP-MDG has increased. For the 4-atom and 7-atom TMNP-MDGs, the addition of dopants strengthens the adsorption energies, while for the 19-atom particles, the addition of nitrogen decreases the O-NP bond strength while the presence of B increases it. In this case, both dopants generate the strongest adsorption energies at the same position of with respect to dopant.

The 4-atom TMNP-MDG experiences an adsorption of O* ~0.25 eV stronger when N is used as a dopant, while the result for B as a dopant only increases by ~0.05 eV. For the 7-atom TMNP-MDG, the addition of a single atom of both N and B implies an increase in the magnitude of adsorption energies by almost 0.2 eV, while adding a second atom barely has any effect; but the quantum-size effect of the 7-atom particle combines to shift the adsorption to ~1 eV weaker than on the 4-atom catalyst in this composition system. Lastly, for the 19-atom particles, the presence of one atom of N increases the adsorption energy by ~0.05 eV while B decreases it about ~0.02 eV. In this case, the addition of second and third dopants has a significant effect, increasing by 0.03 decreases the adsorption of O* by up to 0.05 eV for the nitrogen and decreasing it up to 0.07 eV for the boron.

**Figure 11:**
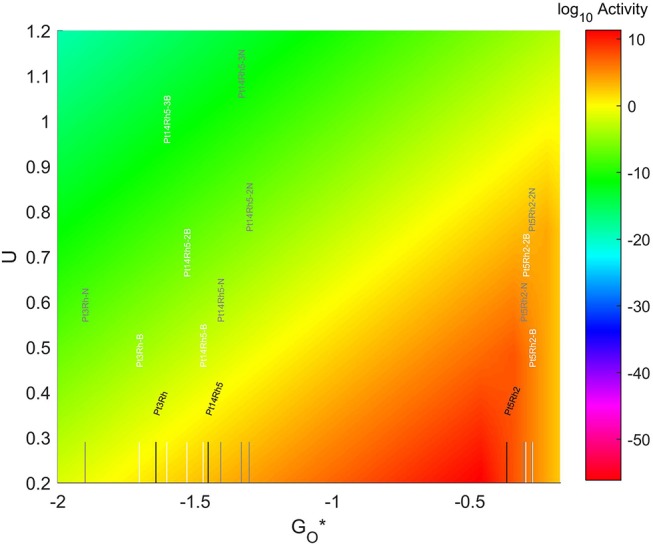
Volcano plot for ORR activity of Pt-Rh 4-atom, 7-atom, and 19-atom TMNP-doped-MDG. 4/7/19 atom systems are Pt_3_Rh_1_, Pt_5_Rh_2_, and Pt_14_Rh_5_, respectively.

The location of these TMNP-MDGs in the volcano plot is shown in Figure 11, zoomed in for visual clarity. The results of the particles supported on pristine graphene have also been included for comparison. The 7-atom TMNP-MDGs are all too oxophobic and end up located on the right side of the predicted volcano maximum. The addition of dopants in the systems with 7 and 19 atoms shifts the location of the alloy TMNP-MDGs toward the right, while doping the systems with 4 atoms shifts them toward the left. The best system in this case is Pt_5_Rh_2_ with no dopants, in its structure, which exceeds the predicted maximum by 0.094 eV.

#### 3.5.4. Rh-Ir TMNP on Doped Graphene

The results for the adsorption energies are included in Figure 15. In all cases the addition of dopants increases the adsorption energy of O*, being the effects much more significant when the dopant is N. The strongest adsorption energies are obtained for the 7-atom particles, which see an increase in magnitude of ~0.25 eV by the inclusion of the N atom in the graphene structure. This increase is only ~0.12 eV when the dopant is B. For the 4-atom particles the increase in O* adsorption is almost 0.4 eV for the systems with N as a dopant, while there is almost no change when the dopant is B. The 19-atom particles see almost no difference by the introduction of B in their structure, while adding N shift the adsorption energy of O* by almost 0.1 eV more weakly. The addition of a second dopant in the 7-atom particles only has a significant effect when the second dopant is B, and it results in a decrease in the adsorption energy by about 0.1 eV. In the 19-atom particles, the addition of a second dopant has an effect if the dopant is N, while the addition of a third dopant only becomes significant if the dopant is B.

**Figure 12:**
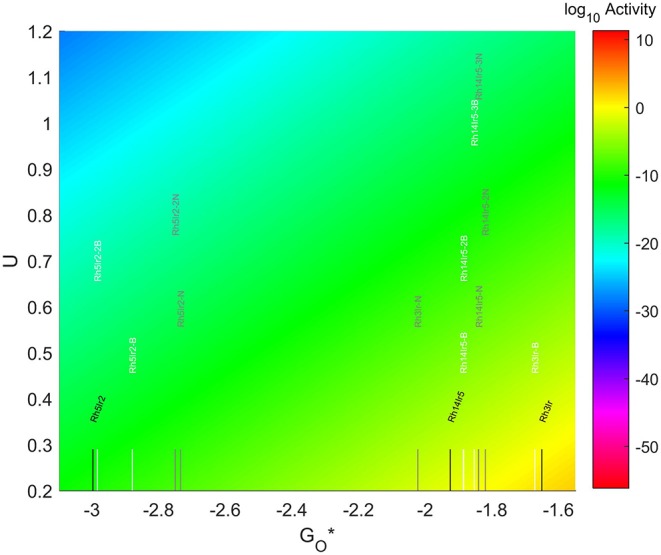
Volcano plot for ORR activity of Rh-Ir 4-atom, 7-atom, and 19-atom TMNP-doped-MDG.4/7/19 atom systems are Rh_3_Ir_1_, Rh_5_Ir_2_, and Rh_14_Ir_5_, respectively.

The location of these nanoparticles in the volcano plot is shown in Figure 12, zoomed in for visual clarity. The results of the particles supported on pristine graphene have also been included for comparison. All the particles in this case, but specially the 7-atom ones, are too oxophilic and appear located on the left of the predicted volcano maximum. In general terms, the addition of dopants shifts the bigger particles (7 and 19-atoms) toward the right, while moves the smallest ones further to the left of the maximum. The best system is Rh_3_Ir with no dopants in its structure, which is located 1.20 eV from the maximum, followed by Rh_3_Ir with one B atom in its graphene structure, which is 1.21 eV from the predicted maximum.

#### 3.5.5. Cu-Ni TMNP on Doped Graphene

The results for the adsorption energies are included in Figure 15. The 4-atom nanoparticles see their O* adsorption energy reduced by the addition of dopants in the graphene structure. The results are especially significant for B, which see a reduction of more than 0.2 eV, while the change for the N-doped system is a reduction in magnitude of 0.05 eV. The opposite occurs for the 7-atom nanoparticles, that see an increase in the magnitude of their O* binding energies when dopants are introduced into the system. This increase is a bit over 0.15 eV for both N and B dopants. The addition of a second dopant in the system decreases the strength of the O-NP bond by about 0.03 eV for the case of N, while increasing it approximately the same amount for the case of B. Thus, the inclusion of a second dopant in the system barely has any effect. For the case of the 19 atom nanoparticles, the inclusion of nitrogen increases the O* binding energies by a very small amount, no matter if one, two, or three nitrogens are added. The most significant change is obtained for the inclusion of 3 nitrogens, with a decrease of binding energy of about 0.02 eV. The addition of boron however, made the adsorption of oxygen weaker, reducing the O* binding energy up to 0.07. This decrease in the binding energy becomes even more significant by the addition of a second and a third dopants, reaching a decrease in the binding energy of up to 0.1 eV for the case with 3 B atoms.

**Figure 13:**
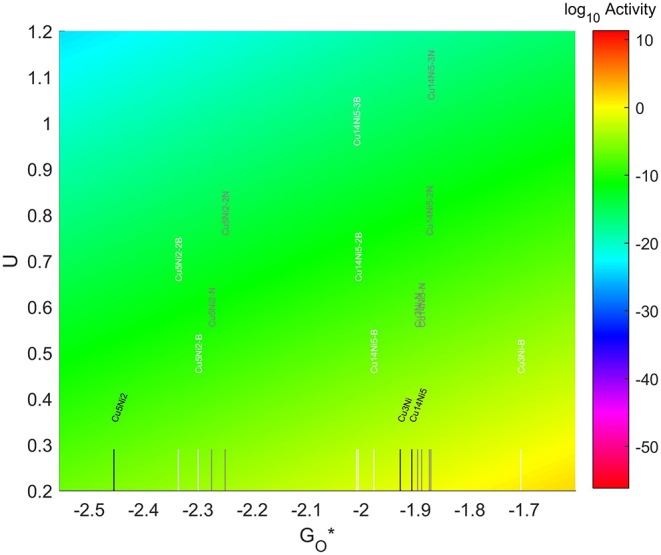
Volcano plot for ORR activity of Cu-Ni 4-atom, 7-atom, and 19-atom TMNP-doped-MDG. 4/7/19 atom systems are Cu_3_Ni, Cu_5_Ni_2_, and Cu_14_Ni_5_, respectively.

The location of these nanoparticles in the volcano plot is shown in Figure 13, zoomed in for visual clarity. The results of the particles supported on pristine graphene have also been included for comparison. While the increase in size makes the particles more oxophilic, resulting in a shift toward the left of the volcano maximum, the introduction of dopants has a significant effect, shifting some of them toward the right and some of them toward the left. In the 7-atom particles the shift is more pronounced in the systems doped with nitrogen, while for the 4-atom and 19-atom particles the largest shift takes place when using B as a dopant. The best system is Cu_3_Ni with one B atom on its graphene structure, which is located 1.28 eV on the left of the predicted volcano maximum. The next best candidate is Cu_14_Ni_5_ supported on graphene doped with 3 atoms of nitrogen, located 1.41 eV on the left of the maximum.

#### 3.5.6. Ni-Cu TMNP on Doped Graphene

The values for the O* adsorption energies can be found in Figure 15. The addition of dopants in the graphene structure results in larger O* adsorption energies for both one and two B dopants and N dopants. When three dopants are introduced, a slight reduction in the O* binding energies is observed.

For the 4-atom particles, the addition of an N dopant makes the O-NP bond 0.17 eV stronger than on the pristine graphene system. This value almost doubles when the dopant used is B instead of N. For the 7-atom particles, adding 1 dopant atom also has a significant effect. When the dopant is N, the O-NP bond becomes 0.18eV stronger than for the case with no dopant, but when B is used the binding energy gets increased by more than 0.4 eV. When adding a second nitrogen, the bond becomes 0.25 eV stronger than for the case with no dopant, but a second boron reduces the strength of the bond by 0.01 eV with respect to the no-dopant system. For the 19-atom particles, introducing an atom of N or B in the graphene structure, makes the O-NP bond 0.04 eV stronger. This shift gets slightly reduced to make the bond 0.03 eV stronger when two dopants are introduced. The presence of a third dopant atom makes the bond weaker than the one seen for no dopants in the system, by 0.01 eV for the case of N and 0.04 eV for the case of B.

**Figure 14:**
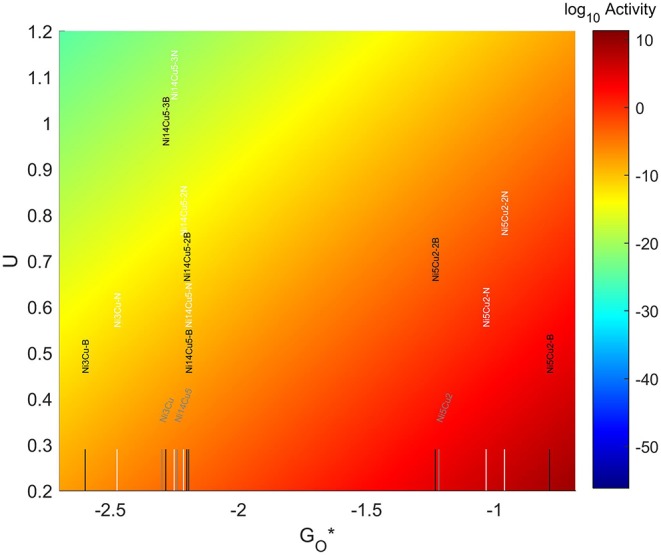
Volcano plot for ORR activity of Ni-Cu 4-atom, 7-atom, and 19-atom TMNP-doped-MDG. 4/7/19 atom systems are Ni_3_Cu, Ni_5_Cu_2_, and Ni_14_Cu_5_, respectively.

The resulting volcano plot is shown in Figure 14, where the location of the systems with pristine graphene has been included for comparison. The addition of dopants into the system makes the 4-atom nanoparticles more oxophilic, shifting them toward the left of the volcano maximum, while making the 7-atom nanoparticles more oxophobic, shifting them toward the right. For the 19 atom nanoparticles, the changes generated by the dopants are not significant enough, and the particles get shift toward the right or the left depending on the dopant and the amount introduced. A significantly good system can be found for a 7-atom system with 1 boron atom in the graphene structure, which misses the predicted maximum by only 0.33 eV to the left.

**Figure 15:**
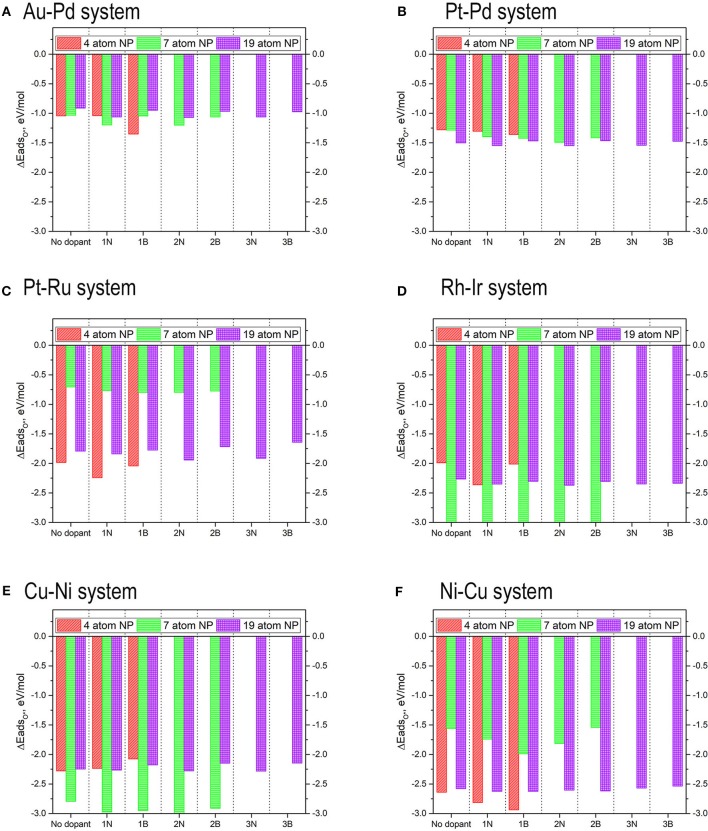
Adsorption energies of mono-atomic oxygen on 4-atom, 7-atom, and 19-atom TMNP-doped-MDG. **(A–F)** identified in Figure, respectively.

### 3.6. Overall Summary of Results for TMNP-MDG for Volcano-Plot Based Optimal Descriptor Values

Based on the information provided in the previous subsections we have observed the following as generalized results in our search for size/composition/support-dopant pairings to shift the mono-oxygen adsorption energy to the predicted volcano peak. First: there is a pronounced non-linear effect in the transition from 4-atom to 7-atom to 19-atom NP which is likely caused by quantum-sized effects such as those seen in recent studies of Pt-based sub-nanometer ORR catalysts (Yamamoto et al., 2009; Imaoka et al., 2013, 2015). This effect significantly destabilizes some of the alloy TMNP-MDG catalysts and makes them fall much closer to the predicted volcano peak than one might expect (Pt/Rh, Ni/Cu, and AuPd). The inclusion of the 7-atom Ni-Cu catalyst system on TMNP-MDiG is promising with respect to our ultimate goal of hopefully engineering ORR catalysts on TMNP-MDG which are free from expensive/rare PMG compositions. Second: the effect of the support-dopant can be very pronounced, contributing to adsorption energy shifts of up to 0.5 eV or more for the O* descriptor. This value however is very specific to the alloy system, location of the dopant with respect to the active site, and does not seem to be further “amplified” by inclusion of additional dopants in the nearby support. This result is promising because the precise synthesis of highly structured/ordered dopants around the catalyst active site beyond a 1-dopant-atom loading might prove difficult if not impossible. Third: the pairing of the correct doping and catalyst composition/size pairing can generate catalyst systems which invert typically expected behavior (of their constituent elements) for ORR activity yet fall remarkably close to the predicted volcano peak. Specifically in this work we have identified candidate TMNP-MDG ORR systems close to the volcano peak which include : Ni_5_Cu_2_-1B, Pt_5_Rh_2_-1N,1B,2N, and Au_14_Pd_5_- 1N,2N,3N. The Au_14_Pd_5_-N system interestingly overbinds O* by only 0.04 eV, and the Pt5-Rh2 system surprisingly underbinds O*. Of most interest and promise however, is the identification of the Ni_5_Cu_2_-1B TMNP-MDG candidate which binds O* more weakly than its constituent elements, and is only ~0.2 eV too oxophilic, a value approximately equivalent to that of the descriptor of Pt(111) on the single crystal ORR volcano. (Nørskov et al., 2004; Gasteiger et al., 2005; Wang C. et al., 2012)

Combining the observations just given, it can be seen that although there are not (yet) any fully identifiable linear mixing rules that predict TMNP-MDG performance across *all* of the range of a 4-atom, 7-atom, or 19-atom for TMNP-MDG catalysts when idealized performance is derived from properties of single elemental constituent species. To the contrary– and most likely due to quantum-sizing effects –intermediately sized 7-atom TMNP-MDG show strongly destabilized oxygen adsorption energies when the constituent metallic species are by themselves strongly oxophilic {7 atom Ni/Cu system discussed in section Ni-Cu TMNP on Doped Graphene, for example}. Despite this observation–it is true that as a general rule—when moving to the 19-atom systems the general trends derived from the constituent elements seem to dictate the performance of the TMNP-MDG catalyst for most of the pairings, with several exceptions including: multiple Boron doped systems, and the Ni-Cu system.

Based on the combined totality of the results presented in our prior work and this manuscript, we recommend future work further probe the non-linear mixing effects in the intermediate sub-nanometer regime, particularly examining compositions including and varying on the 7-atom systems of Ni and Cu (possibly with ternary alloy species such as Pt or Rh), with particle size dependence being treated in parallel up to the 19-atom particle limit already examined in this manuscript (and possibly beyond). Our goal in this work was to examine if there exist composition/size/support-dopant combinations for TMNP-MDG which exhibit oxygen adsorption {the key descriptor} properties that lie near the volcano peak while removing PGM element loading/dependence. Our initial results in this manuscript show that such performance is possible, however it is not yet completely understood how well it can be generalize to related systems; nonetheless we aspire that future work can build on these observations to generate economical, high-performance TMNP-MDG ORR catalysts which remain free of PGM composition.

## 4. Conclusions

In our previous work, a volcano plot for the electrochemical ORR was developed through thermodynamic scaling relations calculated DFT (Lozano and Rankin, 2018; Rankin and Lozano, 2019). Specifically these scaling relations and volcano plots were conducted for subnanometer transition metal intermetallic nanoparticles supported on graphene. Results from the initial investigation confirmed the hypothesis that particles of this size limit—regardless of composition—all bind oxygen too strongly. The predicted maximum catalytic activity was located at –0.46 eV.

In this work, studies on how varying the particle size and including dopants in the graphene structure could shift the predicted activity of different graphene-nanoparticle systems were carried out. Although it appears that their behavior can't be completely predicted by simple linear mixing rules, since both geometric and electronic factors must be taken into consideration, significant shifts were indeed observed for the positions of these particles in the volcano plots and possibly useful catalyst/doped-support pairings with good theoretical ORR performance have been identified.

As a summary of key results, when the size is increased from 4 to 7-atom particles, the best candidate turns out to be Pt_5_Rh_2_, with a binding energy 0.094 eV compared to the predicted maximum. The best 7-atom nanoparticle on the left of the volcano maximum was Pd_2_Au_5_ with a binding energy of -0.693 eV. For the 19-atom nanoparticles, the nanoparticle formed by Au-Pd resulted the best catalyst, missing the predicted maximum by only 0.112 eV on the left side.

The introduction of dopants in the graphene composition affects significantly its electronic structure, introducing a band-gap of up to 1 eV. In turn, this induced indirect bandgap can modulate the electronic structure- and hence- the catalytic activity of the supported TMNP. This results in shifts in the O* adsorption energies of the studied nanoparticles, and in turn, modifies their location in the volcano plots. When including dopants in the graphene structure, the best catalytic systems become the nanoparticles formed by Au-Pd, which bind oxygen too strongly, and the nanoparticles formed with Pt-Rh, which binds too weakly. In particular, Au_14_Pd_5_ adsorbed on graphene with only one N atom, was located at −0.421 eV, missing the maximum by only 0.039 eV, while Pt_5_Rh_2_ with also one N atom ended up at −0.304 eV, only 0.156 eV on the right of the predicted maximum. Thus, the introduction of dopant elements in the graphene support can cause an inversion of the qualitatively expected behavior of the TMNP {Pt/Rh on weakly binding oxophobic leg, Au/Pd on strongly binding oxophilic leg}. The persistence of this unexpected result will be examined in more detail in future work as a function of dopant(s), dopant concentration, particle size, and particle composition.

Further, since none of the studied systems ended up located exactly at the predicted maximum, future studies could be carried out to refine the size and composition of these catalytic systems. In particular, it would be interesting to modify the composition of the 7 and 19 atom nanoparticles by creating ternary alloys, combining through the elements Ni, Cu, Pd, Pt, Au, and Rh, as they have proven to be the ideal metals for the catalysis of ORR at these nanoscales. Additionally, extending the study of dopants to systems with O or S, or even combining more than one dopant in the same structure to balance the acceptor-donor effects, could guide the catalyst to the ideal O* binding energies. Finally, further studies with Ni and Cu, particularly for the 7-atom size, could provide revolutionary knowledge into the ORR catalysts field. Ni and Cu based catalyst would result significantly more affordable than those based on Pt and Rh, making contributions to make fuel cell technologies a reality. Studies with varying composition of Ni and Cu, and even including different dopants in the structure could provide the necessary shifts in O-NP binding energies, to reach the predicted activity maximum in the volcano plot. Finally, with a future extension of this work to include more compositions and particle sizes, it should prove feasible to make a dataset robust enough for machine-learning exercises to generate the predictive design rules for sub-nanometer TMNP-graphene ORR catalyst systems; design of high-performance non-Pt based ORR catalysts may be possible in the near future.

## Data Availability

All datasets generated for this study are included in the manuscript/Supplementary Files.

## Author Contributions

TL performed this research and contributed to writing up the results and discussion presented in this manuscript. RR supervised and helped guide her research efforts and formulated the overall ideas for this manuscript, assisted with writing, and preparation of Figures.

### Conflict of Interest Statement

The authors declare that the research was conducted in the absence of any commercial or financial relationships that could be construed as a potential conflict of interest.
