# Supplementary material for: Size, Composition, and Support-Doping Effects on Oxygen Reduction Activity of Platinum-Alloy and on Non-platinum Metal-Decorated-Graphene Nanocatalysts
Source: Front Chem. 2019 Sep 19;7:610. doi: 10.3389/fchem.2019.00610 (PMC6761360; doi:10.3389/fchem.2019.00610)
Supplement: Supplementary file 1 [file Data_Sheet_1.docx]

**SUPPLEMENTARY INFORMATION**

1. **Tables of DFT energies and O* adsorption energies for 7-atom NP on the different adsorption sites considered**

Cells with yellow background were not calculated because of symmetry. Cells with green background show the positions with the highest energy (weakest bonds). Cells with red background show the positions with the lowest energy (strongest bonds).

1. **Tables of DFT energies and O* adsorption energies for 19-atom NP on the different adsorption sites considered**

Cells with yellow background were not calculated because of symmetry. Cells with green background show the positions with the highest energy (weakest bonds). Cells with red background show the positions with the lowest energy (strongest bonds).

1. **Tables of DFT energies and O* adsorption energies for the different doped systems considered**
2. **Au-Pd systems:** Cells with yellow background show the DFT energies obtained from the calculations. Cells with blue background show the calculated adsorption energy for oxygen. Cells with blue and bold font represent the system with the lowest (strongest) adsorption energy

1. **Pt-Pd systems:** Cells with yellow background show the DFT energies obtained from the calculations. Cells with blue background show the calculated adsorption energy for oxygen. Cells with blue and bold font represent the system with the lowest (strongest) adsorption energy

1. **Pt-Rh systems:** Cells with yellow background show the DFT energies obtained from the calculations. Cells with blue background show the calculated adsorption energy for oxygen. Cells with blue and bold font represent the system with the lowest (strongest) adsorption energy

1. **Rh-Ir systems:** Cells with yellow background show the DFT energies obtained from the calculations. Cells with blue background show the calculated adsorption energy for oxygen. Cells with blue and bold font represent the system with the lowest (strongest) adsorption energy

1. **Cu-Ni systems:** Cells with yellow background show the DFT energies obtained from the calculations. Cells with blue background show the calculated adsorption energy for oxygen. Cells with blue and bold font represent the system with the lowest (strongest) adsorption energy

1. **Ni-Cu systems:** Cells with yellow background show the DFT energies obtained from the calculations. Cells with blue background show the calculated adsorption energy for oxygen. Cells with blue and bold font represent the system with the lowest (strongest) adsorption energy
